# Analyzing sleep status in children with acute leukemia

**DOI:** 10.1186/s13052-023-01409-8

**Published:** 2023-01-14

**Authors:** Lu Xi, Guangsheng Wu, Xinke Du

**Affiliations:** 1 Department of Hematology and Oncology, The Children’s Hospital of Zhejiang University School of Medicine, Zhejiang Pediatric Leukemia Diagnosis and Treatment Technology Center, National Pediatric Health and Disease Clinical Medical Research Center, Hangzhou, 310000 Zhejiang China; 2 Department of Pediatrics, The Affiliated Hospital of Hangzhou Normal University, No. 126 Wenzhou Road, Gongchenqiao Street, Gongshu District, Hangzhou, 310000 Zhejiang China

**Keywords:** Sleep disorders, Scolding, Adenoid hypertrophy, Acute leukemia, Children

## Abstract

**Background:**

Quality sleep is essential for physical and mental health. We aimed to analyze sleep disorders in children with acute leukemia and explore associated factors.

**Methods:**

General data and sleep disorders in children with acute leukemia during chemotherapy were collected by general questionnaires, Children's Sleep Disorders Scale and the Parenting Stress Index-short form.

**Results:**

In total, 173 valid questionnaires were collected. The total Sleep Disorder Scale score > 39 is considered a sleep disorder, while sleep disorders accounted for 45.66% (79/173). In the cohort, 167 children had acute lymphoblastic leukemia, with 40.12% (67/167) having sleep disorders, while six children had acute non-lymphoblastic leukemia, with 50.00% (3/6) having sleep disorders. Single- and multi-factor regression analyses of age, gender, number of children in the family, and time spent using electronic devices showed that factors influencing sleep disorders in these children were mainly parental scolding and adenoid hypertrophy. Children with sleep disorders had more parental stress than those without sleep disorders (*P* < 0.05).

**Conclusions:**

The high incidence of sleep disorders in children with acute leukemia is related to airway conditions and parental behaviors. Sleep disorders in children can increase parenting stress. Factors potentially affecting sleep quality should be addressed as early as possible, while parental education should be strengthened to better facilitate the physical and psychological recovery of their children.

## Introduction

Quality sleep is essential for physical and mental health. Thus, sleep disorders may be associated with impaired cognitive performance [1], mood disorders [2], language and behavior problems like attention deficits [3, 4], lagging performance [5], and obesity [6]. Sleep is influenced by different factors such as genes, brain activity, the sleep environment, and airway conditions [7–9]. The incidence of sleep disorders in children of different ages is high. Difficulty going to bed and frequent waking during the night are reported in 20 to 30 percent of children under 3 years of age and 25 to 50 percent of preschoolers [10–13]. Up to 50% of children having sleep problems [14, 15] and sleep disorders have gained increased research traction. A study reported sleep disturbances in children with leukemia after receiving chemotherapy, but only for a short period of time [16]. There are few studies on long-term sleep and its influencing factors in children with leukemia during chemotherapy. So we hope to assess the long-time sleep situation of leukemia children by sleep disorder scale during the chemotherapy, and collect information like age, electronic products, for analysing of factors affecting the sleep quality in them. We believe our study will comprehensively improve the healthcare management of children with acute leukemia by exploring sleep disorders, identifying associated factors and its relationship with parental stress index. Parenting stress occurs in the process of parenting, and high parenting stress is not conducive to the physical and mental health of parents and children [17]. To understand the parenting status and influencing factors of children with leukemia, and to analyze the correlation between children's sleep disorders and parental parenting stress, so as to provide a basis for reducing parenting stress.

## Materials and methods

### Study information

From 2020 to 2022, the Childhood Sleep Disorders Scale questionnaire was administered to children (and guardians) undergoing chemotherapy in the Department of Hematology Oncology, Children's Hospital of Zhejiang University. General information including age, gender, parents' situation, work status of parents, family monthly income, number of children in the family, place of residence, whether playing cell phones and other electronic products before going to bed, average daily time spent in using electronics, whether scolding children, whether central nervous system involvement(leukemia with central nervous system infiltration), presence or absence of adenoid hypertrophy, was also collected. Inclusion criteria: 1) a confirmed diagnosis of acute lymphoblastic leukemia or acute non-lymphoblastic leukemia; 2) informed consent from guardians. Exclusion criteria: 1) an extremely critical and life-threatening condition; 2) children under the age of 6 months were excluded.

We used the Chinese version of Children's Sleep Disorders Scale (CSDS) made by Bruni et al. [18] and the Parenting Stress Index-short form (PSI-SF) compiled by Abidin [19, 20]*.* The PSI-SF consists of three 12-item subscales, with a total of 36 items, including three dimensions: Parenting distress, parent–child interaction disorder, and difficult child. Each item is rated on a 5-point Likert-type scale with 1 indicating (strongly disagree) and 5 indicating (strongly agree). higher scores were associated with greater stress. The CSDS has high reliability and validity in the context of Chinese culture. The scale consisted of 26 questions divided into six categories: i) difficulty falling asleep and maintaining sleep, ii) sleep breathing disorder, iii) arousal disorder, iv) sleep–wake transition disorder, v) excessive sleepiness, and vi) excessive night sweating. The scale was also divided into five categories: a) none, b) occasionally (< 1–2 times/month), c) sometimes (1–2 times/month), d) often (3 or 5 times/month), and e) always (daily), with scores of 1, 2, 3, 4, and 5, respectively. Higher total scores indicated more severe sleep disorders. A total score > 39 was considered a sleep disorder.

Children’s body mass index (BMI) was calculated by weight (kg)/height squared (m^2^). Children in different age groups were classified as obese, overweight, or lean, with reference to corresponding sex and BMI indices and BMI percentile values for Chinese boys/girls [21]. Children with a BMI in the 85th–95th percentile in the same sex and age group were considered overweight, a BMI > 95^th^ percentile indicated obesity, and a BMI < 3^th^ percentile were considered lean. In this study, children’s BMIs were divided into normal group and abnormal group, with the latter category including obesity, overweight, and lean.

The scale is commonly used to investigate sleep issues in children > 5 years old; Due to several studies suggested the scale was applicable to preschool children and infants [22–24], children < 18 years old were included in this study. Notably, the study mentioned above [22–24] made some adjustments to the scale items and was in diffrent coutries, so we suggest futher research on the application of CSDS in children under 6 years of age in China to increase the validity of the results of this study.

### Statistical analysis

Statistical analyses was performed using SPSS 23.0 software(IBM Corp, Armonk, NY, USA). Chi-square test was used for count data, and t test or analysis of variance was used for measurement data. The factors included age, gender, single-parent family, number of children in the family, parents’ working situation, place of residence, parents' education, monthly household income, playing cell phones and other electronic products before going to bed, average daily time spent in using electronics, scolding children, central nervous system involvement(leukemia with central nervous system infiltration), adenoid hypertrophy. When analysing the influencing factors of sleep disorders, single-factor analysis results showing a significance of *P* < 0.1 were included in multi-factor analysis. Then, multi-factor logistic regression analysis was used for statistical analysis.In multifactor analysis, we considered a *P* value < 0.05 as statistically significant. In the analysis of the influencing factors related to PSI, multiple linear regression analysis was used, with *P* value < 0.05 as statistically significant.

## Results

**Fig. 1:**
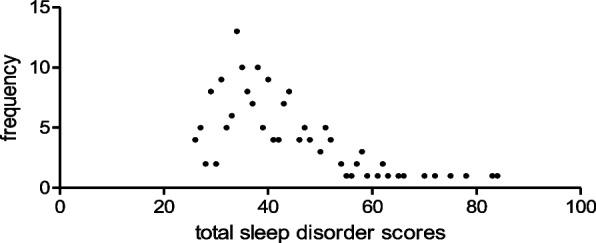
Frequency distribution of total sleep disorder scores

In total, 173 valid questionnaires were collected from children with acute leukemia, with 96 boys and 77 girls. The age range was 8 months–18 years (12 children were 0–2 years, 47 were 3–4 years, 39 were 5–6 years, and 75 were 7–18 years). The mean age and SD was 6.86 ± 3.67. The BMI ranged from 13.10 to 37.12, with a median of 16.80. The total Sleep Disorders Scale score range for all children was 26–84 (Fig. 1), with a median of 38 and interquartile range of 34–46, and 79 cases (45.66%) have sleep disorders. Detailed scores are shown in Table 1.

**Table 1:** The scores of sleep disorders scale

Types of sleep disorders	Total score	minimum	P25	median	P75	maximum
Sleeping difficulties	70	9	9	12	15	15
Sleep breathing disorders	30	3	3	3	4	13
Arousal disorder	30	3	3	3	3	7
Sleep–wake conversion disorder	60	6	8	10	13	28
Excessive sleepiness	50	5	5	5	7	17
Excessive nocturnal sweating	20	2	2	3	5	10
total	260	26	34	38	46	84

Among 173 children, 167 had acute lymphoblastic leukemia, with 40.12% (67/167) having sleep disorders, while six had acute non-lymphoblastic leukemia, with 50.00% (3/6) having sleep disorders. After grouping according to different gender, age and other factors, the situation of sleep disorders in different groups are shown in Table 2. Eleven children had central nervous system leukemia, including seven cases of sleep disorders. The CNS was not involved in 162 children, including 72 cases of sleep disorders. We observed 139 children where electronic devices were used before bedtime, of which 46.04% had sleep disorders (64/139). In 34 children, electronic devices were not used before bedtime, of which 44.12% had sleep disorders (15/34). The sleep disorder rate was 41.07% (23/56) in children who used electronic products for < 3 h/day, 50.00% (33/66) in children who used electronic products for 3–6 h/day, and 45.10% (23/51) in children who used electronic products for > 6 h/day. The rate of sleep disturbance in children in families where parents said they never hit/scolded their children was 35.82% (24/67), and in families where children were scolded, the rate was 51.89% (55/106).

**Table 2:** Sleep disorders in children with acute leukemia

		Total number of cases (a)	Number of cases of sleep disorders (b)	Percentage (b/a*100(%))	χ^2^	*p* value^a^
Sex	Male	96	47	48.96	0.943	0.332
	female	77	32	41.56		
Age	< = 7 years	109	51	46.79	0.150	0.698
	> 7 years	64	28	43.75		
Single Parent	Yes	10	6	60.00	0.879	0.348
	No	163	73	44.79		
Single-child families	Yes (number of children = 1)	65	35	53.85	2.809	0.094
	No (number of children > = 2)	108	44	40.74		
Parents' working situation	Both work	45	19	42.22	1.221	0.543
	Only one works	102	50	49.02		
	Both do not work	26	10	38.46		
Residence	Urban	96	41	42.71	0.760	0.383
	Suburban or rural	77	38	49.35		
BMI	Normal	105	49	46.67	0.108	0.742
	Abnormal (overweight or obese or wasting)	68	30	44.12		
Play cell phones and other electronic products before going to bed	Yes	139	64	46.04	0.041	0.840
	No	34	15	44.12		
Average daily time spent using electronics	< = 3 h	56	23	41.07	0.983	0.612
	3–6 h	66	33	50.00		
	> = 6 h	51	23	45.10		
Scolding children	Never	67	24	35.82	4.271	0.039
	Yes	106	55	51.89		
Central nervous system involvement	Yes	11	7	63.64	1.529	0.216
	No	162	72	44.44		
Adenoid hypertrophy	Yes	8	7	87.5	5.917	0.015
	No	165	72	43.64		

^a^significant test

**Fig. 2:**
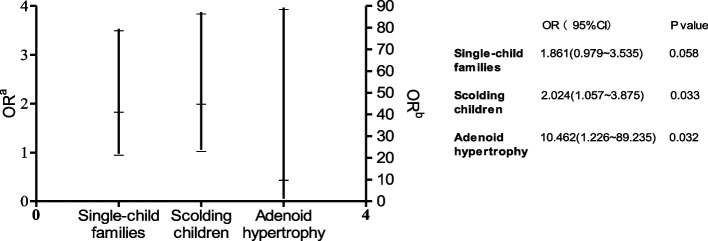
Logistic regression analysis results of factors influencing sleep disorders in children. OR^a^(odds ratio a): OR values of the independent variables single child and scolding are referenced to the left y-coordinate. OR^b^(odds ratio b): OR of the independent variable adenoid hypertrophy is referenced to the y-coordinate on the right

Univariate analyses were performed on gender, age, single parent, single-child family, parental work status, place of residence, BMI, electronic devices used before bedtime, average daily time spent using electronic devices,scolding children, involving the central nervous system, and the presence of adenoid hypertrophy. Univariate analyses showed that child in a single-child family, be beaten/scolded, with adenoidal hypertrophy, were possibly factors related to sleep disturbance in children with acute leukemia (*P* < 0.1, Table 2). Further multifactorial logistic regression analysis suggested that scolding (*P* = 0.033) and adenoid hypertrophy (*P* = 0.032) were risk factors for sleep disturbance in these children (Fig. 2).

The total score of the parenting stress scale was 92.27 for the children with sleep disorder and 82.22 for the children without sleep disorder. In order to understand the influencing factors of parental stress index in children with leukemia, univariate analyses (Table 3) and multiple linear regression analysis were performed on child gender, single parent, the number of child, parental work status, parents' education, number of children, family monthly income, Sleep disorders in children. Through univariate analyses, we found an inverse relationship between parenting stress index and parental education level (*P* < 0.05). In households where both parents were employed and the household monthly income was higher, the parental stress index was lower (*P* < 0.05). Compared to families with children having sleep disorders, parents whose children had sleep disorders had a higher stress index (*P* < 0.05). However, multiple linear regression analysis showed that(F = 5.691, *p* < 0.001), family monthly income (β = -0.233, *P* < 0.007)significantly negatively predicted parental stress index, and children's sleep disorder (β = 0.232, *p* = 0.001)significantly positively predicted parental stress index. These variables explained 16.00% of the variation in parental stress index. Factors such as child gender, single parent, the number of child, parental work status, parents' education, number of children, could not predict the parental stress index. So family monthly income and child with sleep disorders, were both risk factors for PSI.

**Table 3:** Univariate analyses of related factors of Parenting Stress Index(PSI) in children with leukemia

factors		number of cases	mean of PSI	standard deviation	*p* value ^a^
The child gender	male	96	85.80	20.37	0.480
	female	77	88.06	21.62	
single parent family	yes	10	88.80	18.88	0.757
	no	163	86.69	21.07	
Work status of parents	Both have jobs	45	78.47	16.04	0.006
	One has a job	102	89.09	21.70	
	Both have no jobs	26	92.31	21.75	
Parents' education	with a high school diploma or less	83	92.42	21.45	0.000
	Technological academy and above	90	81.39	18.95	
Number of children	one	65	84.38	24.11	0.268
	More than won	108	88.27	18.68	
family monthly income(RMB)	< 5,000	78	93.72	20.95	0.000
	5,000–10,000	49	84.86	21.21	
	> 10,000	46	77.17	16.04	
Sleep disorders in children	yes	79	92.27	20.69	0.001
	no	94	82.22	20.06	

^a^significant test

## Discussion

Sleep disorders that occur in infants often persist into later development [25, 26]. Sleep disorders can impact cognition, mood, social well-being, immunity, and cardiovascular systems [27–29], while high quality sleep enhances immune defenses [30] and allows children with leukemia to sleep well and improve their immunity. Many studies on acute leukemia in children have focused on disease pathogenesis and prognosis, but less on sleep in children with leukemia. Environments where children with acute leukemia undergo chemotherapy are quite different to ordinary environments, therefore factors affecting sleep disorders in these children may be quite different to ordinary children.

From our data, sleep disturbance risk factors in children with acute leukemia during chemotherapy involved parental scolding and adenoid hypertrophy, while age, gender, BMI, single parent families, number of children in the home, and time spent using electronic devices were not risk factors. Previous studies considered single parent and only child families as influencing factors impacting children's sleep [31, 32], but we observed no relationship between these factors, probably because children with leukemia have more time with family and less emotional problems due to parental absence, which laterally suggests relatives help improve children's sleep. We observed no statistically significant differences in total sleep disorder scores of children with leukemia living in urban/suburban areas, which may be related to increased urbanization in China, while no significant differences between urban and suburban environments were observed in Hangzhou, Zhejiang Province, China. A previous study of sleep disorders in children with brain tumors concluded these children were more likely to develop sleep disorders when compared with healthy children [33]. We identified no differences in sleep disorder scores between children with and without CNS leukemia, but the number of cases with definitive CNS leukemia was low, thus these results may be biased. Thus, the impact of CNS leukemia on sleep quality in these children requires further study. Previous studies examined the role of both delayed sleep onset and school demands which reduced sleep levels in adolescents [34]. In China, schoolwork stress significantly increases in children starting at age 7 years, but there was no significant difference in the incidence of sleep disturbance between children aged less than 7 years and those older than 7 years. In this study, presumably due to the fact that children with leukemia during chemotherapy are essentially off school and do not have the need to get up early for school compared to the general population. This suggests the importance of reducing academic pressure and ensuring adequate sleep in school-aged children.

The rate of sleep disturbance was significantly higher in children with leukemia with adenoidal hypertrophy and agreed with previous studies showing that adenoid hypertrophy caused airway hypoventilation and sleep architecture disorders [35]. Studies also reported an increased risk of sleep apnea in obese and underweight children [36, 37]. However, we observed no significant differences in sleep disorders between children with normal and abnormal BMIs, but an insufficient sample size may be partly responsible for this. Additionally, the correlation between BMI and sleep quality may not be significant if BMI abnormalities did not significantly affect airway ventilation conditions during sleep.

Previous studies reported that the longer the exposure time to artificial light and electronic screens, the shorter the sleep time, thus the effects of electronic screens (cell phones and computers) on sleep may be related to psychological and physiological arousal and inhibited melatonin levels [38–40]. However, we observed no effects of electronic product and bedtime use on sleep disorders in children with acute leukemia. We speculate the effects of electronics on sleep may be two-fold. Moderate electronic device use can relax the body and mind, but excessive use at inappropriate times, and an unwillingness to disconnect when the body and mind are in a drowsy state can excite the brain which is not conducive to brain "rest". For children with stable acute leukemia, and for whom chemotherapy is going well, these children are away from school, without schoolwork and accompanied by parents or relatives at home, so their psychological stress is less when compared with healthy children. Their daily use of electronic products is mainly to play simple games, watch short videos or cartoons. Their day and night sleep rhythm is relatively fixed and can basically leave electronic products on time before bedtime under parental supervision. So electronic products have less impact on sleep disorders in children with acute leukemia. We hypothesize that disrupted sleep rhythms exert a larger impact on sleep than staying up late.

Sleep and emotions interact with each other; brain structures and neurochemicals involved in emotion regulation are also involved in sleep [41, 42]. Also, emotional disorders are often accompanied by sleep disturbances [43, 44], while sleep deprivation increases negative emotions but decreases positive emotions [45], and the stronger the negative stimulus, the more sleep is required [46]. Parental scolding can cause sleep disturbance in children with acute leukemia due to negative stimulation caused by parental behaviors.

The current study found that the higher the family income, the lower the parenting stress index, which was also confirmed by Atout M et al. [47]. They also found an association between parental stress and education level, which was not found in this study and may require support from a larger sample size. The biggest innovation of this study is the combination of sleep disorders and parenting stress index in children with leukemia. The decline of children's sleep quality can affect their daytime activities and emotional state, thereby increasing parenting stress. Increased parenting stress also enhances parents' negative emotions, adversely affects parents' physical and mental health, and is not conducive to their care of children. Other studies have found that parenting stress is related to the duration of the disease [48], and the decline of sleep quality in children with leukemia is also related to drug use [49] and fatigue [16]. This study did not count the data of drug use and course of disease in children with leukemia, which is a limitation of this study.

## Conclusions

Adenoid hypertrophy and parental scolding were important factors in sleep disorder development in children with acute leukemia. In the future, we must ensure improved child airway conditions (e.g., prompt treatment of adenoid hypertrophy by hormones or surgery), maintain harmonious parent–child relationships, avoid scolding, and promptly channel children’s negative emotions. In doing so, the sleep quality of children with leukemia can be improved, with their immunity strengthened, parenting stress reduced, and initial steps toward physical and mental health recovery taken.

## Data Availability

The data supporting the results of these studies are available upon reasonable request to the respective authors. The data involving patients are not publicly available due to privacy or ethical restrictions.
